# Canola meal in nursery pig diets: growth performance and gut health

**DOI:** 10.1093/jas/skaa338

**Published:** 2020-10-24

**Authors:** Jinsu Hong, Saymore Petros Ndou, Seidu Adams, Joy Scaria, Tofuko Awori Woyengo

**Affiliations:** 1 Department of Animal Science, South Dakota State University, Brookings, SD; 2 Department of Veterinary & Biomedical Sciences, South Dakota State University, Brookings, SD; 3 Department of Animal Science, Aarhus University, Tjele, Denmark

**Keywords:** canola meal, fecal microbial composition, growth performance, gut permeability, immune response, nursery pigs

## Abstract

An experiment was conducted to determine the effects of including canola meal (**CM**) in nursery pig diets on growth performance, immune response, fecal microbial composition, and gut integrity. A total of 200 nursery pigs (initial body weight = 7.00 kg) were obtained in two batches of 100 pigs each. Pigs in each batch were housed in 25 pens (four pigs per pen) and fed five diets in a randomized complete block design. The five diets were corn–soybean meal (**SBM)**-based basal diets with 0%, 10%, 20%, 30%, or 40% of CM. The diets were fed in three phases: phase 1: day 0 to 7, phase 2: day 7 to 21, and phase 3: day 21 to 42. Diets in each phase were formulated to similar net energy, Ca, and digestible P and amino acid contents. Feed intake and body weight were measured by phase. Immune response and gut integrity parameters were measured at the end of phases 1 and 2. Fecal microbial composition for diets with 0% or 20% CM was determined at the end of phase 2. Overall average daily gain (**ADG**) responded quadratically (*P* < 0.05) to increasing dietary level of CM such that ADG was increased by 17% due to an increase in the dietary level of CM from 0% to 20% and was reduced by 16% due to an increase in the dietary level of CM from 20% to 40%. Pigs fed diets with 0% or 40% CM did not differ in overall ADG. Dietary CM tended to quadratically decrease (*P* = 0.09) serum immunoglobulin A (**IgA**) level at the end of phase 2 such that serum IgA level tended to reduce with an increase in dietary CM from 0% to 20% and to increase with an increase in dietary CM from 20% to 40%. Dietary CM at 20% decreased (*P* < 0.05) the relative abundance of Bacteroidetes phylum and tended to increase (*P* = 0.07) the relative abundance of Firmicutes phylum. Dietary CM linearly increased (*P* < 0.05) the lactulose to mannitol ratio in the urine by 47% and 49% at the end of phases 1 and 2, respectively, and tended to linearly decrease (*P* < 0.10) ileal transepithelial electrical resistance at the end of phase 1 by 64%. In conclusion, CM fed in the current study could be included in corn–SBM-based diets for nursery pigs 20% to improve the growth performance and gut microbial composition and reduce immune response. Also, the CM used in the current study could be included in corn–SBM-based diets for nursery pigs at 30% or 40% without compromising growth performance. Dietary CM increased gut permeability, implying that dietary CM at 20% improves the growth performance of weaned pigs through mechanisms other than reducing gut permeability.

## Introduction

Weaned pigs are highly susceptible to gut infections (Spreeuwenberg et al., 2001; Campbell et al., 2013). Weaning of pigs alters gastrointestinal tract histomorphological characteristics of pigs and induces oxidative stress (Pluske et al., 1997; Hu et al., 2013; Luo et al., 2016), which, in turn, results in increased intestinal permeability (Wijtten et al., 2011) and compromised gut integrity. The increased intestinal permeability leads to increased translocation of toxins that are produced by gut microbiota, causing inflammatory injuries in the gastrointestinal walls or within the body, or both (Lambert, 2009). Most of the infections are caused by inflammatory injury (Wijtten et al., 2011). Thus, the increased susceptibility to gut infections and loss of gut integrity in young pigs during the postweaning period is partly due to damage of intestinal walls, increased intestinal permeability to toxins, and hence inflammatory injury.

The susceptibility to gut infections can be alleviated by reducing oxidative stress or the growth of gut microorganisms that produce toxins. Canola meal (**CM**), which is the second most widely used source of protein in swine diets, has a high content of dietary fiber and glucosinolates (Newkirk et al., 2003; Slominski et al., 2012). Most of the fibers in CM are insoluble (Slominski et al., 2012). Insoluble fiber increases the digesta passage rate, thereby reducing the time for proliferation of pathogenic microorganisms of these microorganisms in the gastrointestinal tract (Becker et al., 2009; Molist et al., 2014). Glucosinolates in canola coproducts exhibited antimicrobial (Aires et al., 2000; Smith et al., 2004; Geng et al., 2011; Dufour et al., 2015) and antioxidant activities in vitro (Wanasundara et al., 1995; Amarowicz and Troszyńska, 2003). Antioxidants reduce immune response (and hence improve health) by reducing oxidative stress (Hoffmann and Berry, 2008; Dalgaard et al., 2018). Thus, it is hypothesized that the inclusion of CM in diets for weaned pigs may improve the gut health by modifying the gut microbial composition and by reducing immune response and injury of intestinal mucosa. However, the effects of including increasing levels of CM in diets for weaned pigs on immune response, gut microbial composition, and gut integrity have not been reported.

In spite of potential positive effects on health, the inclusion of fibrous feedstuffs beyond the optimum level in swine diets can reduce growth performance of pigs by constraining feed intake and lowering energy and nutrient digestibility (Wenk, 2001; Owusu-Asiedu et al., 2006) and by increasing weight of gastrointestinal tract (Stanogias and Pearce, 1985; Nyachoti et al., 2000). Glucosinolates can reduce the growth performance of pigs by reducing feed intake, increasing metabolic activities in the liver, and interfering with thyroid glands function (Tripathi and Mishra, 2007; Mejicanos et al., 2016). Indeed, previous studies have demonstrated that the incorporation of CM in the swine diets may reduce nutrient and energy digestibility (Khajali and Slominski, 2012; Woyengo et al., 2014) and growth performance (Baidoo et al., 1987a, 1987b). Thus, the determination of the effects of including increasing levels of CM in diets for weaned pigs on immune response, gut microbial composition, gut integrity, and integration of this information with that on the effects of dietary CM on growth performance and visceral organ weights and function is critical for identifying the optimal level of inclusion of CM in diets for weaned pigs.

The objective of this study was to determine the effects of including CM in diets for nursery pigs on growth performance; visceral organ weights; blood concentration of thyroid hormones, immunoglobulins, and proinflammatory cytokine; fecal microbial composition; small intestinal histomorphology; gastrointestinal tract permeability; and electrophysiological parameters of small intestine mounted in Ussing chambers.

## Materials and Methods

Experimental procedures were reviewed and approved by the Institutional Animal Care and Use Committee at South Dakota State University (Experiment Protocol number: 18-040A).

### Animals and housing

A total of 200 twenty-one-day-old pigs (initial body weight [**BW**] of 7.00 ± 1.02 kg; Large White-Landrace female × Large White-Hampshire male; Pig Improvement Company) were obtained from Swine Education and Research Facility, South Dakota State University (Brookings, SD) in two batches of 100 pigs immediately after weaning. Pigs in each batch were housed in 25 pens: 15 pens of 4 barrows and 10 pens of 4 gilts in batch 1 and 10 pens of 4 barrows and 15 pens of 4 gilts in batch 2. Pens (1.0 × 1.0 × 1.0 m) had metallic floor (fully slated), walls, and gates. Each pen was equipped with a water nipple, a three-spaced dry feeder, and a heat lamp. Room temperature was maintained at 28 ± 1 °C during the first 1 wk. Thereafter, the room temperature was maintained at 27 ± 1 °C throughout the experiment.

### Experimental diets

Five corn–soybean meal (**SBM**)-based diets containing 0%, 10%, 20%, 30%, or 40% CM were offered to the nursery pigs throughout the experiment (Table 1). The CM fed in this study was sourced from South Dakota State University’s feed mill in a single batch and had been produced by the prepress solvent extraction method. The diets were formulated to meet the NRC (2012) nutrient recommendations for nursery pigs. The diets were fed as a mash for 6 wk in three phases: phase 1: day 0 to 7, phase 2: day 7 to 21, and phase 3: day 21 to 42. Diets for each phase were formulated to provide similar net energy, Ca, standardized total tract digestible P, and standardized ileal digestible amino acid contents.

**Table 1.** Ingredient composition of experimental diets, as-fed^1^

	Phase 1					Phase 2					Phase 3				
	CM inclusion level, %					CM inclusion level, %					CM inclusion level, %				
Item	0	10	20	30	40	0	10	20	30	40	0	10	20	30	40
Ingredient, % as-fed															
Corn	53.56	49.65	46.89	44.85	36.85	57.73	54.05	49.80	49.05	43.33	66.71	65.60	63.70	61.57	54.44
SBM	24.00	17.84	10.51	2.50	—	26.04	19.62	13.71	4.50	—	29.70	20.83	10.70	4.80	1.70
CM	—	10.00	20.00	30.00	40.00	—	10.00	20.00	30.00	40.00	—	10.00	20.00	30.00	40.00
Whey permeate	10.00	10.00	10.00	10.00	10.00	10.00	10.00	10.00	10.00	10.00	—	—	—	—	—
Soy protein^2^	3.00	3.00	3.00	3.00	3.00	2.00	2.00	2.00	2.00	2.00	—	—	—	—	—
Fish meal	5.00	5.00	5.00	5.00	5.00	—	—	—	—	—	—	—	—	—	—
Soybean oil	1.06	1.36	1.56	1.66	2.60	0.41	0.69	1.04	0.99	1.51	0.10	0.00	0.17	0.27	0.95
Limestone	0.92	0.84	0.73	0.66	0.56	1.19	1.10	1.03	0.94	0.83	1.16	1.06	0.98	0.91	0.81
Monocalcium phosphate	0.68	0.57	0.54	0.48	0.37	0.98	0.90	0.83	0.78	0.72	0.89	0.87	0.82	0.74	0.61
l-Lysine·HCl	0.51	0.53	0.58	0.66	0.56	0.53	0.55	0.56	0.67	0.64	0.41	0.57	0.59	0.67	0.59
dl-Methionine	0.10	0.10	0.10	0.11	0.10	0.12	0.12	0.12	0.14	0.13	0.09	0.10	0.11	0.12	0.10
l-Threonine	0.16	0.15	0.15	0.16	0.10	0.15	0.14	0.13	0.15	0.12	0.12	0.14	0.16	0.15	0.11
l-Tryptophan	0.02	0.02	0.04	0.05	0.04	0.00	0.01	0.02	0.04	0.04	0.00	0.01	0.03	0.04	0.03
Salt	0.79	0.74	0.70	0.67	0.62	0.65	0.62	0.56	0.54	0.48	0.62	0.58	0.54	0.51	0.46
Vitamin premix^3^	0.05	0.05	0.05	0.05	0.05	0.05	0.05	0.05	0.05	0.05	0.05	0.05	0.05	0.05	0.05
Mineral premix^4^	0.15	0.15	0.15	0.15	0.15	0.15	0.15	0.15	0.15	0.15	0.15	0.15	0.15	0.15	0.15
Calculated provisions^5^															
Crude fat, %	3.96	4.82	5.58	6.29	7.69	2.96	3.83	4.51	5.18	6.33	2.96	3.50	4.17	4.93	6.03
CP, %	21.61	22.29	22.60	22.66	22.78	20.21	20.79	21.61	21.25	22.52	20.20	19.94	19.98	20.08	22.25
Net energy, Mcal/kg	2.498	2.498	2.498	2.498	2.500	2.461	2.461	2.461	2.461	2.461	2.430	2.430	2.430	2.430	2.435
NDF, %	6.99	8.75	10.52	12.29	13.98	7.49	9.25	11.00	12.79	14.52	8.52	10.31	12.09	13.86	15.58
SID^6^ Lys, %	1.50	1.50	1.50	1.50	1.50	1.35	1.35	1.35	1.35	1.35	1.23	1.23	1.23	1.23	1.23
SID Lys/NE, g/Mcal	0.60	0.60	0.60	0.60	0.60	0.548	0.548	0.548	0.548	0.548	0.506	0.506	0.506	0.506	0.506
SID Met, %	0.43	0.43	0.43	0.43	0.43	0.39	0.39	0.39	0.39	0.39	0.36	0.36	0.36	0.36	0.36
SID Thr, %	0.88	0.88	0.88	0.88	0.88	0.79	0.79	0.79	0.79	0.79	0.73	0.73	0.73	0.73	0.74
SID Trp, %	0.25	0.25	0.25	0.25	0.25	0.22	0.22	0.22	0.22	0.22	0.21	0.20	0.20	0.20	0.20
Total Ca, %	0.85	0.85	0.85	0.85	0.85	0.80	0.80	0.80	0.80	0.80	0.70	0.70	0.70	0.70	0.70
STTD^7^ P, %	0.45	0.45	0.45	0.45	0.45	0.40	0.40	0.40	0.40	0.40	0.33	0.33	0.33	0.33	0.33

^1^The experimental diets were fed in three phases: phase 1 from day 0 to 7, phase 2 from day 7 to 21, and phase 3 from day 21 to 42.

^2^Soy protein was a hydrolyzed soy protein product (HP 300) from Hamlet Protein (Horsens, Denmark).

^3^Provided the following per kilogram of diet: 11,011 IU vitamin A, 1,652 IU vitamin D_3_, 55 IU vitamin E, 0.04 mg vitamin B_12_, 4.4 mg menadione, 9.9 mg riboflavin, 61 mg pantothenic acid, 55 mg niacin, 1.1 mg folic acid, 3.3 mg pyridoxine, 3.3 mg thiamine, and 0.2 mg biotin.

^4^Provided the following per kilogram of diet: 165 mg Zn as ZnSO_4_, 23 mg Fe as FeSO_4_; 17 mg Cu as CuSO_4_, and 44 mg Mn as MnSO_4_.

^5^Calculated nutrient content was based on ingredient composition data from NRC (2012).

^6^SID, standardized ileal digestible.

^7^STTD, standardized total tract digestible.

### Experimental design and procedure

The five diets were allotted to the 25 pens used in each block (five pens per diet) in a randomized complete block design for a total of 10 replicates per diet in the entire study. Feed and fresh water were provided to pigs all the times during the experimental period. The BW of the pigs and feed intake were measured at the beginning of the study and at the end of each phase to calculate average daily gain (**ADG**), average daily feed intake (**ADFI**), and gain-to-feed (**G:F**) ratio. At the end of phases 1 (day 7) and 2 (day 21) of feeding, one pig (per pen) with a BW that was close to the average BW of pigs in that particular pen was selected for measuring blood parameters, gut permeability and histomorphology, and electrophysiological parameters of small intestine mounted in Ussing chambers. Due to the limited number of chambers that we had, the selected pigs were sacrificed at the rate of five pigs (balanced for diet) per day from day 7 to 11 and from day 21 to 25 of each batch. Three hours prior to euthanization, a bolus (15 mL/kg) of 5% lactulose and 5% d-mannitol solutions was orally administered to pigs that had been marked for euthanization. Also, prior to euthanization, 10 mL of blood was collected from each pig via jugular vein puncture into two sets of blood serum tubes coated with silica (BD Vacutainer, Plymouth, UK; 5 mL/tube). The collected blood was centrifuged at 1,872 × *g* for 20 min at 4 °C to recover serum, which was stored frozen at −20 °C for latter determination of serum triiodothyronine (**T3**), tetraiodothyronine (**T4**), immunoglobulin G (**IgG**), immunoglobulin A (**IgA**), immunoglobulin M (**IgM**), and tumor necrosis factor-alpha (**TNF-α**) concentrations. The pigs were anesthetized by an intramuscular mixture of telazol-ketamine-xylazine (telazol and xylazine at 50 mg/mL each; ketamine at 100 mg/mL). After the abdomen was numbed with lidocaine, a small incision was made in the lower abdomen, which allowed access to the bladder. The urine sample was collected directly from the bladder using a sterile needle and syringe. The collected urine samples were snap-frozen in liquid nitrogen and stored at −80 °C for later determination of gut permeability by the lactulose:mannitol ratio in urine. After blood and urine collection, the anesthetized pig was euthanized by exsanguination. Upon euthanasia, the following procedures took place. Ten-centimeter sections of the jejunum (at the middle of small intestine) and ileum (at 70 cm above ileocecal junction) were immediately collected and placed in ice-cold Ringer’s solution (NaCl; 6.72 g/L, K_2_HPO_4_; 0.42 g/L, KH_2_PO_4_; 0.05 g/L, CaCl_2_ dihydrate; 0.18 g/L, MgCl_2_ hexahydrate; 0.24 g/L, NaHCO_3_; 2.1 g/L, glucose 1.80 g/L; pH of 7.3 to 7.4) for the determination of intestinal electrophysiological properties using an Ussing chambers technique as described below. Indomethacin (10 µM) was added into the buffer solution at 3 µL/L to help minimize the effects of proinflammatory eicosanoids on intestinal tissue electrophysiological properties. Also, 5-cm sections of the duodenum (at 70 cm below the pylorus) and of jejunum and ileum (from the same locations where sections for the Ussing chamber were obtained) were obtained and placed into 10% buffered formalin solution for later determination of histology. The pig was eviscerated to harvest the liver, kidneys, spleen, heart, and thyroid gland. Each of these organs was isolated, blot dried, and weighed. The stomach, small intestine, cecum, and colon were also collected, emptied, blot dried, and weighed. Fresh fecal sample was collected directly from each pig’s rectum after euthanization of pigs from days 21 to 25. The collected fecal samples were immediately snap-frozen using liquid N and stored at −80 °C for later analysis of microbial composition.

### Sample preparation and analyses

Experimental diets and CM were ground to pass through a 0.75-mm screen using a centrifugal mill (model ZM200; Retsch GmbH, Haan, Germany). The ground CM and diet samples were analyzed for dry matter (**DM**), crude protein (**CP**), ether extract (**EE**), crude ash, acid detergent fiber (**ADF**), neutral detergent fiber (**NDF**), and glucosinolates. The samples were analyzed for DM by oven drying at 135 °C for 2 h (method 930.15), CP by a combustion procedure (method 990.03), EE (method 2003.06), and crude ash (method 942.05) as per AOAC (2007), and for ADF and NDF (Van Soest et al., 1991) on an Ankom 200 Fiber Analyzer (Ankom Technology, Fairport, NY). The glucosinolate content was quantified by gas chromatography (POS Pilot Plant Corp., Saskatoon, SK, Canada) according to the method of Daun and McGregor (1981).

The serum concentration of T3 was determined using an immunoassay analyzer (Immulite 1000, DPC, Los Angeles, CA), whereas serum T4 concentration was determined using Clinical Chemistry Auto-Analyzer System (Vet Axcel Chemistry Analyzer, Alfa Wassermann Diagnostic Technologies, West Caldwell, NJ). The serum concentration of IgA, IgG, and IgM was determined by the enzyme-linked immunosorbent assay (**ELISA**) according to the manufacture’s guidelines (Pig IgA ELISA Kit, Pig IgG ELISA Kit, Pig IgM ELISA Kit; Bethyl Laboratories, Inc., Montgomery, TX). The serum concentration of TNF-α was determined by the ELISA assay according to the manufacture’s guideline (TNF alpha Porcine ELISA Kit, Invitrogen, Carlsbad, CA).

For analysis of the fecal microbial composition, total microbial DNA was extracted from fecal samples using the PowerFecal Pro DNA kit (QIAGEN, MD, US) following the manufacturer’s instructions. The quality of the DNA was determined using NanoDrop one (Thermo Fisher Scientific, DE) and quantified using Qubit Fluorometer 3.0 (Invitrogen, CA). The extracted DNA samples were used for the sequencing of the hypervariable V3-V4 regions of the bacterial 16S rRNA using the Illumina MiSeq platform. The library preparation for metagenomic sequencing was performed using 0.3 ng of DNA with a Nextera XT library preparation kit (Illumina, San Diego, CA) and sequenced on the MiSeq Platform. The variations in bacterial communities within the feces of nursery pigs were analyzed using 16S rRNA microbial community analysis package in Quantitative Insights into Microbial Ecology framework (QIIME, Version 2.0). Briefly, 33 samples were quality filtered, demultiplexed, and denoised using dada2. The outputs were transferred to R for analysis using phyloseq. Shannon diversity index and Simpson diversity index were used to estimate the α-diversity index. The taxonomy was assigned to amplicon sequence variants using the dada2 package to implement the naive Bayesian classifier method against GreenGenes (http://greengenes.lbl.gov). The operating taxonomic units (OTUs) were clustered with 97% similarity cutoff using USEARCH and Chimeric sequences, subsequently filtered out to obtain OTUs for species classification. The percentage abundance of each taxa representing the phylum was plotted using Explicit v2.10.5. The sequences have been deposited into the NCBI database, accession number PRJNA664381.

Intestinal tissue samples for histology analysis were sent to the Animal Disease Research and Diagnostic Laboratory at South Dakota State University (Brookings, SD) for staining with hematoxylin and eosin. Villous height (**VH**; from the top of the villi to the villous-crypt junction) and crypt depth (**CD**; from the villous-crypt junction to the base) were measured at 4× magnification using a microscope (Micromaster, Fisher Scientific, Waltham, MA, USA) equipped with a 0.55× wifi camera eyepiece (MC500-W 3rd Gen., Meiji Techno Co. LTD., Saitama, Japan) and Micro-Capture software (Meiji Techno Co. LTD., Saitama, Japan) in 20 well-oriented villi and crypt columns. The VH:CD ratio was calculated.

Gastrointestinal tract permeability was quantified by the ratio of lactulose:mannitol concentrations in the urine. The concentrations of lactulose and mannitol in urine were determined by ELISA assay (BioAssay System, Hayward, CA). The 96-well plates for lactulose assay were prepared and incubated in the darkness for 60 min at room temperature, whereas well plates for mannitol assay were prepared and incubated in light for 30 min at room temperature before reading optical density at 565 nm.

The electrophysiological properties (potential difference [**PD**], short-circuit current [**Isc**], and transepithelial electrical resistance [**TEER**]) were determined using an Ussing chamber (VCC-MC6; Physiologic Instruments Inc., San Diego, CA, US) containing pairs of current (Ag wire) and voltage (Ag/AgCl pellet) electrodes housed in 3% agar bridges and filled with 3 M KCl. Samples for determining gut permeability were transported (while in ice-cold Ringer buffer solution) to the laboratory, where they were opened along the mesenteric border. The opened samples were gently stripped of the serosal layer using micro-forceps to remain with the mucosa. The prepared mucosal tissues (three tissues per intestinal segment) were placed in tissue holders with an aperture of 1 cm^2^. The first slider of the tissue holder was placed over the serosal side of the tissue. The second slider of the tissue holder was placed on the mucosal side of the tissue, and two sliders were gently pressed together. Liquid was dried out from chamber recess, and the tissue holders were mounted in the chambers with the mucosal layer facing the left side of the chamber. The 3 mL of 3 M KCl solution was added to the serosal and mucosal half chambers, respectively. The chambers were continuously gassed with a mixture of 95% O_2_ and 5% CO_2_. The temperature of the chambers was maintained at 37 °C. After mounting tissues in the chambers, the Ussing chamber system was placed in remote mode to allow for Acquire and Analyze software program to obtain data, and tissues were referenced on the Acquire and Analyze Data acquisition software and hardware system. After referencing, 3 µL of d-glucose (10 µM) was added to the serosal bathing solution, which was balanced on the mucosal side with 3 µL d-mannitol (10 µM). The Acquire and Analyze software was turned on immediately after adding glucose and mannitol to start collecting data, and then tissues were allowed to equilibrate for about 15 min. After the equilibration, 30 mM of d-glucose was added to the mucosal side and balanced with 30 mM of d-mannitol of the serosal side for measuring the glucose-induced Isc. After observing peak Isc values due to the addition of glucose on the mucosal side of chambers, the tissues were allowed to equilibrate for 10 to 15 min. Thereafter, TEER and Isc were recorded for 60 mins for the determination of intestinal permeability. After 60 min, 3 µL of forskolin (10 µM) was added to both the mucosal and serosal sides of the cambers to determine whether or not the tissues were still alive. Significant changes in Isc within 10 min indicated that the tissues were still alive. Glucose-induced Isc (active transport of glucose across the mucosa) was calculated as the difference between the Isc values (basal Isc values) that were recorded at the time of adding 30 mM glucose on the mucosal side and 30 mM mannitol on the serosal side and the greatest Isc values that were recorded after the addition of 30 mM glucose on the mucosal side and 30 mM mannitol on the serosal side. 

### Statistical analysis

Data were analyzed using the MIXED procedure of SAS (ver. 9.4, SAS Institute Inc., Cary, NC) in a randomized complete block design with pen as the experimental unit. The model included diet as the fixed effect and batch and block as the random effects. Linear and quadratic contrasts for equally spaced levels were performed to determine the effects of increasing inclusion of CM in nursery diets. Initial BW was used as a covariate for growth performance data. To test the hypotheses, *P* < 0.05 was considered significant. If pertinent, trends (0.05 ≤ *P* ≤ 0.10) are also reported.

## Results

The analyzed CP content in the diets (Table 2) was similar to the calculated CP content in the diets (Table 1). The increasing inclusion level of CM increased the NDF, ADF, and total glucosinolates contents in the diets. The total glucosinolates content of CM used in the current study was 6.15 µmol/g (Table 3). Progoitrin was the most abundant aliphatic glucosinolate, whereas 4-hydroxyglucobrassicin was the most abundant aromatic glucosinolate present in CM fed in the current study.

**Table 2.** Analyzed chemical composition of experimental diets (as-fed basis)^1^

	Phase 1					Phase 2					Phase 3				
	CM, %					CM, %					CM, %				
Item	0	10	20	30	40	0	10	20	30	40	0	10	20	30	40
DM, %	87.69	87.29	88.80	87.45	88.96	86.85	87.96	87.56	86.85	86.72	88.08	85.14	84.89	85.56	86.83
CP, %	21.28	22.10	21.95	21.08	24.58	18.67	18.11	20.70	19.25	21.01	18.78	18.75	17.83	17.93	20.47
Crude ash, %	5.77	6.47	6.57	6.27	6.75	5.95	5.86	6.06	5.85	5.73	5.60	5.38	4.67	4.80	5.09
EE, %	2.77	3.60	4.04	4.43	5.36	2.00	2.18	2.88	2.94	3.40	1.61	1.67	2.03	2.48	2.62
NDF, %	6.51	8.78	10.47	11.09	13.16	6.40	8.32	10.20	10.75	12.09	7.85	9.65	10.76	12.44	14.81
ADF, %	4.15	6.03	6.55	8.35	9.61	4.41	5.26	6.98	7.41	8.76	4.65	5.26	7.83	8.25	8.99
Total glucosinolates, µmol/g^2^	0.00	0.62	1.23	1.85	2.46	0.00	0.62	1.23	1.85	2.46	0.00	0.62	1.23	1.85	2.46

^1^The experimental diets were fed in three phases: phase 1 from day 0 to 7, phase 2 from day 7 to 21, and phase 3 from day 21 to 42.

^2^Calculated values based on analyzed value (6.15 µmol/g) of total glucosinolates in CM.

**Table 3.** Analyzed nutrient and glucosinolates content of CM (as fed-basis)

Item	CM
Nutrient composition, %	
Moisture	11.78
CP	37.47
Crude ash	6.48
EE	3.53
NDF	22.48
ADF	18.40
Glucosinolates^1^, µmol/g	
Gluconapin (3-Butenyl)	1.50
Glucobrassicanapin (4-Pentenyl)	0.16
Progoitrin (2-OH-3-Butenyl)	2.59
Gluconapoleiferin (2-OH-4 Pentenyl)	0.09
Total aliphatics	4.34
Glucoerucin (4-CH_3_-Sulfinyl-Butyl)	<0.02
Gluconasturitiin (Phenethyl)	0.06
Glucoberteroin (5-CH_3_-Sulfinyl-Pentyl)	0.63
Glucobrassicin (3-Indolymethyl)	0.16
4-Hydroxyglucobrassicin (4-OH-3-Indolylmethyl)	0.96
Total aromatics	1.81
Total glucosinolates	6.15

^1^Glucosinolate values were analyzed by gas chromatography (Daun and McGregor, 1981) at POS Pilot Plant Corp. (Saskatoon, SK, Canada).

Increasing dietary CM level from 0% to 40% quadratically affected (*P* < 0.10) BW at the end of phase 1 and ADG for phase 1 such that BW and ADG of pigs numerically increased with increase in the dietary level of CM from 0% to 20% and then reduced (*P* < 0.05) due to an increase in the dietary level of CM from 20% to 40% (Table 4). The BW of pigs at the end of phases 2 and 3, and ADG and ADFI of pigs for phase 2 and for the entire study responded quadratically (*P* < 0.05) to dietary increase in CM such that these response criteria were increased (*P* < 0.05) when dietary CM was increased from 0% to 20% and then decreased (*P* < 0.05) when dietary CM was increased to 40%. Increasing dietary inclusion of CM from 0% to 40% quadratically affected (*P* < 0.05) G:F of pigs for phase 1 such that G:F was increased (*P* < 0.05) when dietary CM was increased from 0% to 20% and then decreased (*P* < 0.05) when dietary CM was increased to 40%. The G:F for phase 2 and the overall G:F were linearly increased (*P* < 0.05) with the incremental levels of dietary CM.

**Table 4.** Growth performance of nursery pigs fed increasing levels of CM

	CM, %						*P*-value		
Item	0	10	20	30	40	SEM	Diet	Linear	Quadratic
BW, kg									
Initial	6.98	7.02	6.86	7.12	6.99	0.846	0.881	0.993	0.973
Day 7	7.17^ab^	7.28^ab^	7.33^a^	7.19^ab^	7.10^b^	0.107	0.247	0.439	0.084
Day 21	10.92^b^	12.11^a^	12.20^a^	11.57^ab^	11.04^b^	0.265	0.001	0.731	<0.001
Day 42	25.10^b^	26.57^ab^	27.64^a^	26.36^ab^	25.28^b^	0.722	0.079	0.949	0.007
ADG, g/d									
Day 0 to 7	27^ab^	43^ab^	52^a^	30^ab^	16^b^	16.9	0.261	0.447	0.093
Day 7 to 21	268^b^	345^a^	347^a^	314^ab^	282^b^	16.3	0.003	0.936	<0.001
Day 21 to 42	675	689	736	704	678	28.9	0.557	0.824	0.148
Overall	323^b^	359^a^	378^a^	352^ab^	325^b^	17.5	0.007	0.940	<0.001
ADFI, g/d									
Day 0 to 7	85	90	95	79	75	8.9	0.460	0.248	0.222
Day 7 to 21	372^b^	421^a^	437^a^	355^b^	337^b^	21.0	<0.001	0.023	0.001
Day 21 to 42	1,108	1,171	1,193	1,097	1,106	79.2	0.467	0.616	0.230
Overall	521^bc^	561^ab^	572^a^	519^abc^	501^c^	18.0	0.022	0.136	0.007
G:F ratio									
Day 0 to 7	0.181^ab^	0.462^ab^	0.560^a^	0.490^ab^	0.141^b^	0.185	0.136	0.994	0.016
Day 7 to 21	0.728^b^	0.813^ab^	0.812^ab^	0.876^a^	0.846^a^	0.060	0.038	0.010	0.211
Day 21 to 42	0.611	0.594	0.617	0.659	0.629	0.037	0.677	0.329	0.955
Overall	0.495^b^	0.622^ab^	0.663^a^	0.637^ab^	0.562^ab^	0.057	0.173	0.375	0.017

^a–c^Within a row, means without a common superscript differ (*P* < 0.05).

There were no effects of dietary level of CM on the weights of thyroid gland, liver, kidneys, spleen, cecum, or large intestine relative to live BW at the end of phase 1 (Table 5). An increase in the dietary level of CM from 0% to 40% resulted in a linear increase (*P* < 0.05) in the heart weight relative to live BW at the end of phase 1. Increasing dietary CM level from 0% to 40% tended to quadratically affect (*P* < 0.10) the weight of small intestine relative to live BW at the end of phase 1 such that the relative small intestine weight tended to be increased (*P* < 0.10) when dietary CM was increased from 0% to 20% and then decreased (*P* < 0.05) when dietary CM was further increased to 40%. At the end of phase 2 of the study, dietary CM linearly increased (*P* < 0.05) the thyroid gland weight relative to live BW and linearly decreased (*P* < 0.05) the heart weight or small intestine weight relative to live BW. Also, the liver weight relative to live BW tended to be linearly increased (*P* = 0.08) by the increasing dietary inclusion of CM. Dietary CM did not affect the relative weights of kidneys, spleen, stomach, cecum, or colon.

**Table 5.** Organ weights relative to live BW of nursery pigs fed increasing levels of CM

	CM, %						*P*-value		
Organ weight, g/kg BW	0	10	20	30	40	SEM	Diet	Linear	Quadratic
Phase 1									
Thyroid gland	0.093	0.093	0.104	0.091	0.098	0.011	0.755	0.717	0.624
Heart	4.52	5.18	5.20	5.02	5.73	0.225	0.060	0.036	0.311
Liver	24.86	25.13	25.35	23.41	26.88	0.890	0.143	0.411	0.203
Kidneys	4.89	5.17	5.05	4.99	5.36	0.249	0.588	0.275	0.771
Spleen	2.94	3.09	3.03	3.23	2.61	0.238	0.419	0.488	0.159
Stomach	8.04	8.07	7.91	7.43	7.55	0.347	0.565	0.129	0.890
Small intestine	37.83^ab^	37.77^ab^	43.14^a^	33.47^b^	34.03^b^	2.016	0.011	0.067	0.069
Cecum	2.37	2.12	2.50	2.44	2.09	0.188	0.291	0.645	0.326
Colon	13.25	12.93	14.58	12.59	12.53	1.157	0.497	0.532	0.429
Phase 2									
Thyroid gland	0.110	0.122	0.147	0.144	0.165	0.012	0.002	<0.001	0.794
Heart	5.41	5.24	5.15	5.18	4.89	0.159	0.253	0.035	0.839
Liver	30.59	31.74	33.54	33.57	33.43	1.785	0.335	0.080	0.340
Kidneys	5.74	5.08	5.50	5.60	5.33	0.186	0.152	0.614	0.520
Spleen	2.49	2.07	2.70	2.30	2.52	0.327	0.601	0.749	0.806
Stomach	9.61	8.72	9.91	9.32	9.28	0.542	0.684	0.963	0.428
Small intestine	52.80	50.11	45.59	48.17	44.75	3.456	0.060	0.028	0.635
Cecum	2.61	2.67	2.56	2.77	2.55	0.197	0.884	0.999	0.611
Colon	19.38	16.85	16.93	19.13	18.44	1.721	0.648	0.995	0.386

^a,b^Within a row, means without a common superscript differ (*P* < 0.05).

The serum T3 and T4 concentrations at the end of phase 1 were not influenced by dietary inclusion of CM (Table 6). At the end of phase 2 of the study, incremental levels of dietary CM linearly increased (*P* < 0.05) the serum T3 concentration and tended to linearly decrease (*P* = 0.05) the serum T4 concentration. There were no effects of dietary level of CM on serum IgA, IgG, IgM, and TNF-α concentrations of pigs at the end of phase 1 (Table 7). Dietary CM tended to quadratically affect (*P* = 0.09) the serum IgA concentration at the end of phase 2 such that serum IgA concentration tended to reduce with an increase in the dietary level of CM from 0% to 20% and then tended to increase with a further increase in dietary CM to 40%; the serum IgA concentration for diet with 0% CM did not differ from that of diet with 40% CM. The serum IgG, IgM, and TNF-α concentrations were not affected by the dietary inclusion level of CM at the end of phase 2.

**Table 6.** Serum thyroid hormone levels of nursery pigs fed increasing levels of CM

	CM, %						*P*-value		
Item	0	10	20	30	40	SEM	Diet	Linear	Quadratic
Phase 1									
T3, ng/dL	68.2	72.9	66.3	81.8	82.7	11.80	0.797	0.302	0.750
T4, mg/dL	3.10	3.31	3.08	2.99	3.09	0.308	0.906	0.524	0.962
Phase 2									
T3, ng/dL	121.9	102.7	165.5	150.0	148.1	17.20	0.014	0.032	0.432
T4, mg/dL	3.22	2.83	2.74	2.37	2.42	0.314	0.277	0.052	0.582

**Table 7.** Serum immunoglobulins and cytokine of nursery pigs fed increasing levels of CM

	CM, %						*P*-value		
Item	0	10	20	30	40	SEM	Diet	Linear	Quadratic
Phase 1									
IgA, ng/mL	129.6	123.6	133.2	150.5	128.8	16.35	0.837	0.686	0.732
IgG, ng/mL	7,326	6,314	7,042	6,017	7,173	849.4	0.777	0.930	0.486
IgM, ng/mL	478.7	585.2	559.5	319.4	447.4	401.50	0.241	0.586	0.550
TNF-α, pg/mL	240.1	273.3	215.7	214.7	222.0	117.93	0.119	0.361	0.682
Phase 2									
IgA, ng/mL	276.3^ab^	289.4^ab^	232.5^b^	256.0^ab^	306.5^a^	24.58	0.246	0.702	0.098
IgG, ng/mL	5,642	5,268	5,161	5,160	3,921	1,780.9	0.456	0.671	0.414
IgM, ng/mL	511.8	433.0	514.8	466.2	475.4	340.50	0.853	0.758	0.957
TNF-α, pg/mL	146.5	193.6	129.2	139.5	138.7	87.44	0.110	0.689	0.884

^a,b^Within a row, means without a common superscript differ (*P* < 0.05).

**Figure 1.**
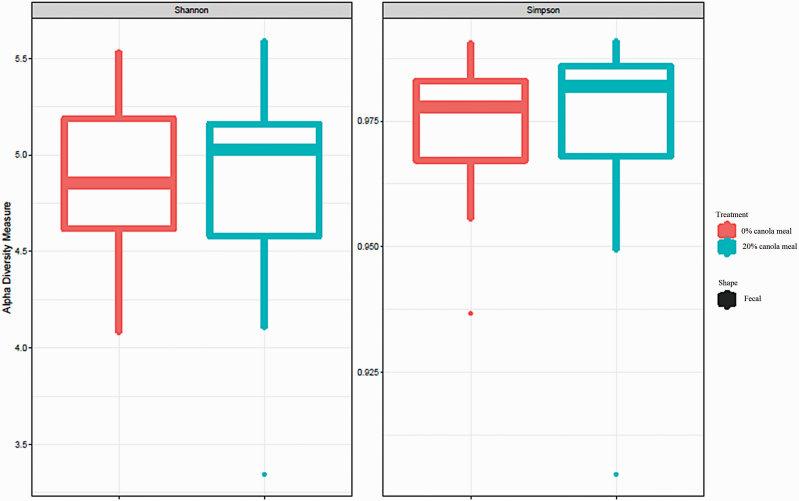
Alpha diversity indices (Shannon diversity index and Simpson diversity index) of fecal microbiome of pigs fed diets with 0% and 20% of CM.

**Figure 2.**
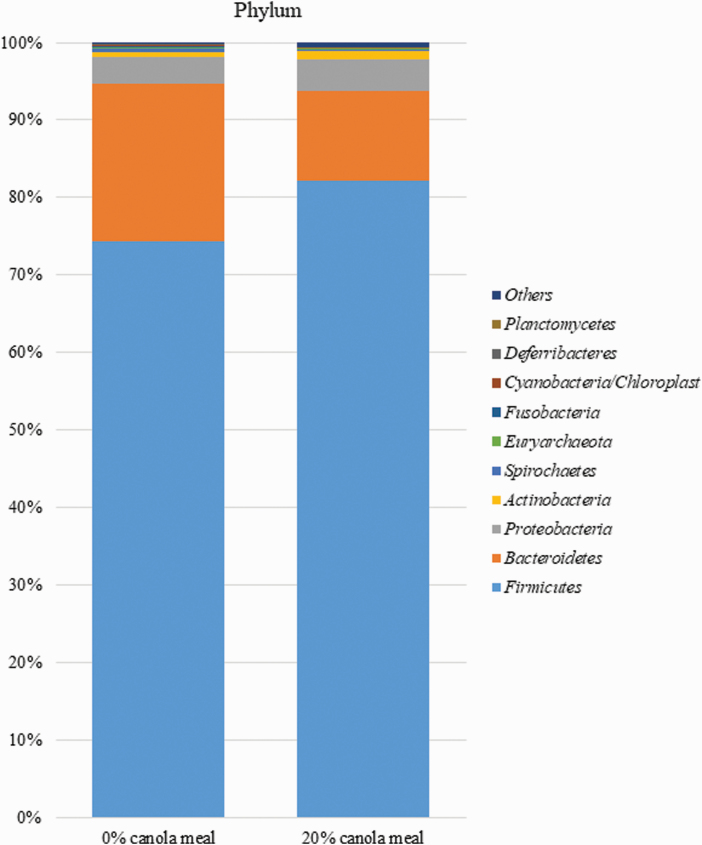
The distribution of bacterial communities at the phylum level of pigs fed diets with 0% and 20% of CM.

**Figure 3.**
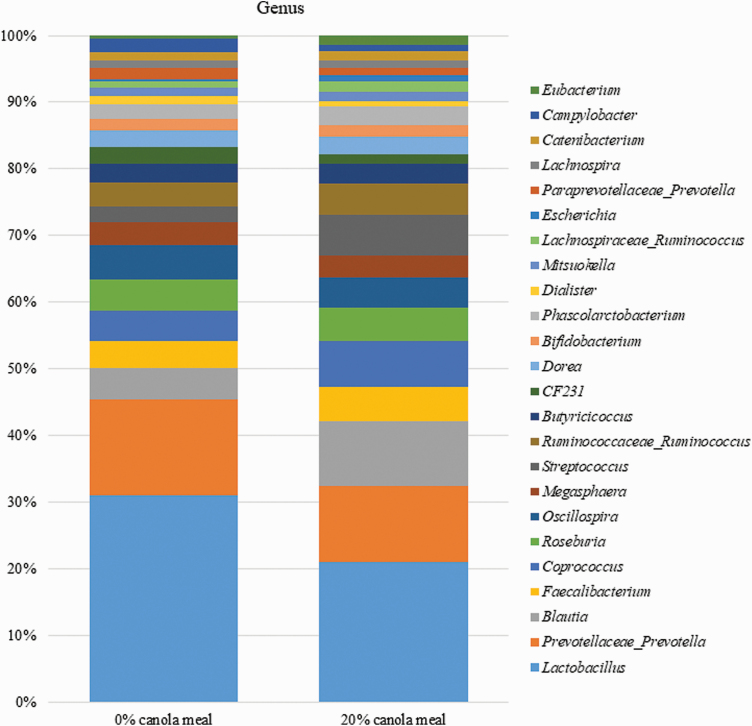
The distribution of bacterial communities at the genus level of pigs fed diets with 0% and 20% of CM.

The inclusion of CM in diets at 20% did not affect the diversity indexes of the fecal microbiome compared with those of control diet (Figure 1). The relative abundance of Bacteroidetes at the phylum level for CM 0% was higher (*P* < 0.05) than that for CM 20% (Table 9; Figure 2). Also, the inclusion of CM in diets at 20% tended to increase (*P* = 0.07) the relative abundance of Firmicutes phylum and to decrease (*P* = 0.10) the relative abundance of Cyanobacteria/Chloroplast phylum (Table 8). However, the dietary inclusion level of CM did not affect the abundance of other microorganisms, including Proteobacteria, Actinobacteria, and Spirochaetes at the phylum level. Inclusion of CM in diets at 20% increased (*P* < 0.05) the relative abundance of *Blautia* and *Eubacterium* genera that belong to Firmicutes phylum (Figure 3). The dietary inclusion level of CM did not influence the abundance of other microorganisms including *Lactobacillus*, *Prevotella*, *Faecalibacterium*, *Coprococcus*, *Roseburia*, *Oscillospira*, *Megasphaera*, *Streptococcus*, *Ruminococcus*, and *Butyricicoccus* at the genus level.

**Table 8.** Relative abundance of bacteria at phylum level in the feces of nursery pigs

	CM, %			
Phylum	0	20	SEM	*P*-value
Firmicutes	74.40	82.16	2.904	0.069
Bacteroidetes	20.25	11.64	2.172	0.009
Proteobacteria	3.46	3.95	1.303	0.793
Actinobacteria	0.70	1.10	0.246	0.250
Spirochaetes	0.43	0.25	0.122	0.298
Euryarchaeota	0.21	0.19	0.067	0.802
Fusobacteria	0.16	0.00	0.077	0.157
Cyanobacteria/Chloroplast	0.12	0.00	0.048	0.099
Deferribacteres	0.08	0.00	0.039	0.166
Planctomycetes	0.06	0.08	0.040	0.702

**Table 9.** Small intestinal histology of nursery pigs fed increasing levels of CM

	CM, %						*P*-value		
Item	0	10	20	30	40	SEM	Diet	Linear	Quadratic
Phase 1									
Duodenum									
VH, μm	354.9	348.8	357.0	366.9	323.7	96.38	0.452	0.562	0.622
CD, μm	297.9	279.8	323.6	302.8	272.9	26.72	0.241	0.558	0.233
VH to CD ratio	1.19	1.24	1.11	1.22	1.20	0.237	0.665	0.839	0.811
Jejunum									
VH, μm	354.0	365.9	389.5	369.6	338.3	74.76	0.384	0.667	0.384
CD, μm	223.4	237.9	257.8	242.5	242.4	25.69	0.502	0.392	0.431
VH to CD ratio	1.57	1.54	1.53	1.51	1.42	0.188	0.743	0.222	0.766
Ileum									
VH, μm	293.9	317.9	313.2	322.8	315.0	78.69	0.834	0.835	0.775
CD, μm	200.4	217.0	224.9	223.2	252.3	14.73	0.159	0.021	0.776
VH to CD ratio	1.52	1.56	1.44	1.44	1.28	0.138	0.665	0.182	0.593
Phase 2									
Duodenum									
VH, μm	353.8	385.9	387.6	387.2	376.2	40.45	0.671	0.507	0.351
CD, μm	249.6	262.1	257.6	252.3	270.8	27.40	0.871	0.511	0.838
VH to CD ratio	1.44	1.48	1.52	1.57	1.42	0.085	0.802	0.857	0.322
Jejunum									
VH, μm	368.2	347.9	383.2	338.5	307.0	30.57	0.008	0.026	0.212
CD, μm	233.2	234.2	223.0	245.4	227.8	25.94	0.679	0.918	0.761
VH to CD ratio	1.58	1.50	1.75	1.43	1.39	0.088	0.048	0.115	0.148
Ileum									
VH, μm	296.6	313.0	335.6	320.9	308.1	26.02	0.376	0.442	0.175
CD, μm	200.4	185.5	203.8	198.3	195.6	16.59	0.561	0.681	0.847
VH to CD ratio	1.52	1.72	1.66	1.64	1.61	0.094	0.661	0.786	0.253

The CD of the ileum at the end of phase 1 was linearly increased (*P* < 0.05) with the increasing level of dietary CM from 0% to 40% (Table 9). The VH in jejunum at the end of phase 2 was linearly decreased (*P* < 0.05) due to an increase in dietary CM level. The dietary inclusion of CM had no influence on the VH and CD in the duodenum. An increase in the dietary level of CM linearly increased (*P* < 0.05) the lactulose:mannitol ratio at the end of phase 1 (Table 10). At the end of phase 1, the dietary inclusion of CM from 0% to 40% linearly increased (*P* < 0.05) the lactulose:mannitol ratio in the urine.

**Table 10.** Lactulose and mannitol concentration and lactulose to mannitol ratio in urine of nursery pigs fed increasing levels of CM

	CM, %						*P*-value		
Item	0	10	20	30	40	SEM	Diet	Linear	Quadratic
Phase 1									
Lactulose, mM	2.59	2.65	2.26	2.43	3.03	0.597	0.797	0.509	0.411
Mannitol, mM	20.15	19.63	18.64	16.62	13.51	2.850	0.463	0.099	0.573
Lactulose:mannitol	0.134	0.143	0.121	0.228	0.259	0.047	0.147	0.041	0.347
Phase 2									
Lactulose, mM	0.76	0.77	0.97	0.87	0.93	0.266	0.899	0.468	0.923
Mannitol, mM	25.70	33.54	29.45	25.47	18.38	6.369	0.102	0.074	0.040
Lactulose:mannitol	0.034^b^	0.024^b^	0.044^ab^	0.037^ab^	0.066^a^	0.019	0.080	0.032	0.149

^a,b^Within a row, means without a common superscript differ (*P* < 0.05).

Data on the effect of dietary inclusion of CM on electrophysiological properties for the jejunum and ileum are presented in Table 11. An increase in the dietary level of CM from 0% to 40% did not affect jejunal basal Isc, glucose-induced ΔIsc, TEER, and average Isc (Isc that was recorded when TEER was recorded). The ileal TEER at the end of phase 1 tended to decrease linearly (*P <* 0.10) due to an increase in the dietary level of CM. The ileal average Isc at the end of phase 1 was linearly increased (*P* < 0.05) as dietary CM increased. Conversely, incremental levels of dietary CM did not influence the ileal TEER and average Isc at the end of phase 2.

**Table 11.** Electrophysiological properties (Isc and TEER) of the small intestine of nursery pigs mounted in Ussing chambers

	CM, %						*P*-value		
Item	0	10	20	30	40	SEM	Diet	Linear	Quadratic
Phase 1									
Jejunum									
Basal Isc, μA/cm^2^	14.05	10.88	8.97	9.44	8.10	10.891	0.995	0.711	0.883
Glucose induced ΔIsc, μA/cm^2^	−1.06	−1.05	−1.01	−1.97	−0.88	2.450	0.999	0.972	0.977
Average Isc^1^, μA/cm^2^	17.46	16.80	15.81	18.73	20.48	9.203	0.997	0.788	0.799
TEER, Ω · cm^2^	38.10	29.77	34.91	31.06	40.86	10.083	0.749	0.541	0.483
Ileum									
Basal Isc, μA/cm^2^	−5.99	9.31	28.93	−2.64	29.14	12.720	0.191	0.175	0.703
Glucose induced ΔIsc, μA/cm^2^	5.41	3.89	6.21	8.66	3.77	10.141	0.995	0.926	0.957
Average Isc, μA/cm^2^	8.80	14.12	10.94	15.93	44.22	12.181	0.160	0.035	0.217
TEER, Ω · cm^2^	39.03	29.19	27.95	23.73	13.95	10.540	0.473	0.097	0.939
Phase 2									
Jejunum									
Basal Isc, μA/cm^2^	2.90	8.41	−2.25	5.80	17.49	7.236	0.100	0.347	0.471
Glucose induced ΔIsc, μA/cm^2^	−0.11	−0.44	−0.21	0.18	0.15	2.577	0.989	0.717	0.836
Average Isc, μA/cm^2^	10.14	18.25	37.34	17.10	25.64	12.531	0.453	0.498	0.398
TEER, Ω · cm^2^	63.99	65.01	55.74	45.92	45.54	12.585	0.704	0.178	0.913
Ileum									
Basal Isc, μA/cm^2^	2.25	0.24	3.25	6.61	3.31	6.872	0.976	0.714	0.932
Glucose induced ΔIsc, μA/cm^2^	0.52	0.21	1.20	0.51	0.26	0.912	0.940	0.848	0.135
Average Isc, μA/cm^2^	13.04	4.36	11.77	6.50	6.43	5.997	0.716	0.584	0.789
TEER, Ω · cm^2^	51.07	41.28	49.54	39.05	51.40	14.238	0.950	0.973	0.645

^1^Average Isc = average of Isc values that were recorded when TEER values were also recorded.

## Discussion

The analyzed contents of CP, ash, EE, and NDF in CM were comparable to the values reported by NRC (2012) and Parr et al. (2015) for the CM produced by the prepress solvent extraction method. The total glucosinolate content of CM (6.15 µmol/g) fed in the current study was within the range of values (1.11 to 8.7 µmol/g) that were reported by others (Landero et al., 2011; Parr et al., 2015; Wang et al., 2017) for prepress solvent-extracted CM. The NDF content of CM (22.5%) was also within the range of values (21.5% to 27.2%) that were reported by others (Bell and Keith, 1991; Zhou et al., 2013; Woyengo et al., 2016) for solvent-extracted CM. In general, the growth of pigs is positively related to voluntary feed intake or nutrient intake. Thus, the quadratic response of ADG to increasing dietary inclusion of CM observed in the current study could partly be attributed to the quadratic response of ADFI to increasing dietary inclusion of CM. Canola coproducts contain glucosinolates that can reduce the growth performance of pigs. Pigs can tolerate up to 2.50 µmol of total glucosinolate per gram of diet before feed intake and BW gain becomes impaired (Woyengo et al., 2017). In the current study, the calculated total glucosinolate content in diets containing 0%, 10%, 20%, 30%, and 40% CM were 0, 0.62, 1.23, 1.85, and 2.46, respectively. Thus, the lack of difference between the diet containing 0% CM and diet containing 40% CM with regard to ADG and ADFI could be attributed to the fact that the total glucosinolate content in diet containing 40% CM was within the tolerable level. Similarly, Landero et al. (2011) reported that an increase in the dietary level of total glucosinolate from 0 to 0.77 µmol/g through an increase in the dietary level of CM from 0% to 20% did not affect the growth performance of nursery pigs. Parr et al. (2015) observed that an increase in the dietary level of total glucosinolate from 0 to 3.48 µmol/g through an increase in the dietary level of CM from 0% to 40% did not affect the ADG, but linearly reduced the ADFI of nursery pigs.

An increase in the relative abundance of Firmicutes in the gastrointestinal tract resulted in an increase in BW of pigs (Guo et al., 2008; Pedersen et al., 2013) and humans (Ley et al., 2006; Indiani et al., 2018) and was partly attributed to increased efficiency of digestion and absorption of energy in the gastrointestinal tract (Hildebrandt et al., 2009; Wang et al., 2019). Lee (2019) observed that an increase in dietary inclusion of cold-pressed canola expellers from 0% to 40% resulted in the relative abundance of Firmicutes in the gastrointestinal tract of weaned pigs. Thus, the increase in the relative abundance of Firmicutes due to dietary inclusion of CM at 20% observed in the current study could partly explain the increase in ADG of pigs fed diet with 20% CM. However, the ADG results from the current study are in contrast to those from the study of Lee (2019) who observed reduced ADG of weaned pigs due to dietary inclusion of cold-pressed canola expellers at 20% or 40%. Notably, the cold-pressed canola expellers fed in the study of Lee (2019) had greater content of glucosinolates than the CM fed in the current study (14.9 vs. 6.15 µmol/g), implying that the level of glucosinolates in the cold-pressed canola expellers-containing diets fed in the study of Lee (2019) exceeded the tolerance level. Thus, it appears that in the study of Lee (2019), the positive effects of dietary cold-pressed canola expellers on the gastrointestinal tract microbial composition were partly countered by the negative effects of the same feedstuff on voluntary feed intake and liver and kidney functions due to the greater content of glucosinolates in the diets. The results from the current study are also in contrast with those from the study of Landero et al. (2011) who reported similarity between weaned pigs fed diet with 0% CM and those fed diet with 20% CM with regard to ADG. Wang et al. (2017) reported an increase in ADG and ADFI of weaned pigs due to the replacement of 20% CM from one source with 20% CM from another source in wheat-based diets. These specific two CM fed in the study of Wang et al. (2017) contained less than 5.0 µmol/g (4.67 vs. 2.10 µmol/g) total glucosinolates and did not differ in CP (37.3% vs. 36.2%), EE (3.5% vs. 3.8%), and total dietary fiber (20.0% vs. 22.9%). Thus, it appears that there are factors other than total glucosinolates content that might influence the effect of dietary CM on the growth performance of weaned pigs.

The increased relative weights of the liver and thyroid gland at the end of phase 2 of the study due to dietary CM may be explained by the increased metabolic activity in these organs. Glucosinolate degradation products such as goitrin impair iodine uptake by the thyroid gland, leading to increased metabolic activity in the thyroid gland and hence enlarged thyroid gland (Sihombing et al., 1974; Schöne et al., 1997; Tripathi and Mishra, 2007). Glucosinolate degradation products are detoxified in the liver and hence an increase in the consumption of glucosinolate-containing feedstuffs can lead to increased activity of detoxification enzymes in hepatic tissues, thereby increasing the relative weight of the liver (Munday and Munday, 2002; Nho and Jeffery, 2001; Tanii et al., 2005). Parr et al. (2015) and Lee and Woyengo (2018) also reported increased relative weights of thyroid glands and liver of nursery pigs due to an increase in the dietary level of canola coproducts from 0% to 40%. In the current study, the increased serum T3 level and reduced serum T4 level due to dietary CM may be explained by impairment of iodine uptake in the thyroid gland by dietary glucosinolates. Impaired iodine uptake by thyroid glands results in reduced synthesis of T4 by the thyroid glands, thereby leading to reduced blood T4 concentration and hence increased production of thyroid-stimulating hormone by the pituitary gland (Rousset et al., 2015). An increase in thyroid-stimulating hormone production can result in increased conversion of T4 into T3 in tissues other than the thyroid gland, leading to increased blood concentration of T3 (Rousset et al., 2015). Results from the current study are in agreement with those from the studies of Parr et al. (2015) and Lee and Woyengo (2018), who reported reduced serum level of T4 in nursery pigs due to an increase in the dietary level of canola coproducts. In the current study, dietary CM did not affect the relative weights of thyroid glands and liver, and serum concentration of T3 and T4 at the end of phase 1 of the study, which could be attributed to the fact that the feeding period (7 d) was not long enough for dietary CM to affect these response criteria.

The relative weight of the heart was increased at the end of phase 1 and reduced at the end of phase 2 due to the dietary inclusion of CM. Woyengo et al. (2011) similarly reported an increase in the heart weight due to dietary inclusion level of expeller-extracted CM from 0% to 40% in broilers. However, the mechanisms by which dietary CM could affect the relative weight of the heart have not been well established. Thus, there is a need to establish the mechanisms by which dietary CM affect the relative weight of the heart in weaned pigs.

The relative weight of the small intestine at the end of phase 1 was unaffected by an increase in the inclusion of CM in diets from 0% to 20% but was reduced by an increase in the dietary level of CM from 20% to 40%. At the end of phase 2, the relative weight of small intestine was linearly reduced by an increase in the dietary level of CM from 0% to 40%. Dietary fiber, whether fermentable or unfermentable, is positively associated with small intestinal weight (McCullogh et al., 1998). CM has higher fiber content than SBM that it replaced in the control diet. Thus, it is unclear why the relative weight of the small intestine was reduced by dietary CM. Fermentation of fiber in the hindgut may promote the proliferation of beneficial intestinal microbiota with a high capacity to produce short-chain fatty acids including butyric acid (Freire et al., 2000; Molist Gasa et al., 2010). Butyric acid is used as a source of energy by colonocytes (Montagne et al., 2004; Hedemann and Knudsen, 2007). Thus, the weight of the hindgut is expected to increase with an increase in fiber content in diets. However, CM fiber is highly lignified (Khajali and Slominski, 2012) and hence less fermentable than that in SBM (Woyengo et al., 2016). Thus, the lack of effect of dietary CM on relative weights of cecum and large intestine in the current study could be attributed to the fact that fiber in CM is less fermentable.

Immunoglobulin A is an immunoglobulin that is secreted by mucosa to protect the mucosa from infections (Cerutti et al., 2011; Corthésy, 2013). In the gastrointestinal tract, IgA binds to dietary antigens and proinflammatory bacterial epitopes, thereby protecting the gastrointestinal tract mucosa from infections (Lamm et al., 1995; Fagarasan and Honjo, 2003; Snoeck et al., 2006). The production of IgA is induced by the presence of feed or microbial antigens (Van den Broeck et al., 1999; Sun et al., 2008), and hence the production of IgA is related to the amount of antigens and composition of microorganisms in the gastrointestinal tract (Gutzeit et al., 2014; Macpherson et al., 2015; Boyaka, 2017). Thus, the decrease in serum IgA concentration due to an increase in the dietary level of canola from 0% to 20% and an increase in serum IgA concentration (to a level similar to that observed for diet with 0% CM) due to an increase in dietary CM from 20% to 40% imply that dietary inclusion of CM at 20% reduced immune response. Serum IgA concentration was reduced by decrease in relative abundance of Proteobacteria in the gastrointestinal tract (D’auria et al., 2013; Wilmore et al., 2018). In the current study, dietary CM did not affect the relative abundance of Proteobacteria in feces. Thus, mechanisms by which dietary CM at 20% reduced serum IgA need to be elucidated. Also, mechanisms by which serum IgA concentration increased (to the level for a diet with 0% CM) by increasing the level of dietary CM from 20% to 40% are not clear. Dietary CM did not significantly affect systemic IgG, IgM, and TNF-α production as evidenced by a lack of effect of dietary CM on serum concentration of the aforementioned immune parameters. Lee and Woyengo (2018) and Parr et al. (2015) reported that supplementation of CM did not affect the white blood cell, neutrophil, lymphocyte, and monocyte in blood. Glucosinolates in CM have antioxidant and antimicrobial activities. Feed additives that have antioxidant activity or antimicrobial activity or both can affect immune parameters including proinflammatory cytokines (IL-1 and IL-18), anti-inflammatory cytokines (IL-6 and IL-10) and oxidative stress biomarkers (superoxide dismutase, glutathione peroxidase, nicotinamide adenine dinucleotide phosphate oxidase, and xanthine oxidase) that were not measured in the current study locally (within the gastrointestinal tract) or within the whole body.

Based on the growth performance, the dietary level of CM for weaned pigs that resulted in maximal BW and ADG was 20%. Thus, diet with 20% CM was chosen for determining the effects of dietary inclusion of CM on fecal microbial composition. Firmicutes and Bacteroidetes are the most abundant phyla in the gut microbiota of pigs and can produce volatile fatty acid from small intestinal undigested carbohydrates and protein in the hindgut of pigs (Leser et al., 2002; Bhandari et al., 2008). The Bacteroidetes and Firmicutes are heavily affected by dietary fiber (Molist et al., 2012; Deehan et al., 2018). A higher ratio of Firmicutes to Bacteroidetes promoted by dietary fiber has been reported to reduce diarrhea and incidence of infectious and inflammatory diseases (Mulder et al., 2009; Middelbos et al., 2010). Molist et al. (2012) observed increased relative abundance of Firmicutes and reduced relative abundance of Bacteroidetes in the colonic digesta and feces of weaned pigs due to dietary inclusion of coarse-ground wheat bran but not fine-ground wheat bran. Fouhse et al. (2017) observed an increased relative abundance of Firmicutes in the ileal digesta of weaned pigs due to the dietary inclusion of resistant starch. In the current study, *Blautia* and *Eubacterium* are the genera of microorganisms under Firmicutes phylum whose relative abundance in feces was increased by increasing the dietary level of CM from 0% to 20%. The gastrointestinal tract *Eubacterium* was positively correlated with body weight of children (Sepp et al., 2013); gastrointestinal fermentation of dietary fiber, particularly resistant starch, increased the relative abundance of *Eubacterium* in feces of humans (Martínez et al., 2010; Venkataraman et al., 2016). With regard to *Bluatia,* their relative abundance in feces was increased by the utilization of resistant starch as a substrate for microorganisms (Yang et al., 2013). Also, increased consumption of whole grains such as barley and brown rice increased the relative abundance of *Blautia* in the feces of humans (Martínez et al., 2013). The CM has a greater content of fiber than SBM; however, CM fiber (compared with SBM fiber or resistant starch) is poorly fermented because it is highly lignified. Thus, it is not clear whether or not the increase in the relative abundance of *Blautia* and *Eubacterium* and hence Firmicutes observed in the current study was due to fiber present in CM. Furthermore, in some human studies, the relative abundance of Firmicutes was increased by replacing fibrous diets with Western-type diets that have low fiber and high fat content (De Filippo et al., 2010; Amato et al., 2015). Thus, there is a need to establish the mechanisms by which dietary CM increased the relative abundance of Firmicutes in the gastrointestinal tract of pigs.

The VH in the small intestine of weaned pigs is positively related to feed intake because of the increase in small intestinal luminal nutrient availability with the increase in feed intake (Pluske et al., 1996). However, in the current study, the linear decrease in jejunal VH at the end of phase 2 due to the dietary inclusion of CM may not be explained by a change in feed intake because ADFI responded quadratically to dietary inclusion of CM. The corn and SBM that CM replaced in the control diet have a greater content of digestible carbohydrates (starch for corn and sucrose for SBM) than the CM (NRC, 2012). Also, the dietary level of soybean oil, which increased with increase in the dietary level of canola, does not contain carbohydrates. Partial replacement of glucose plus glutamine with soybean oil as a source of energy for intestinal epithelial cells resulted in reduced VH of the small intestine in piglets (Yang et al., 2016). Thus, in the current study, the reduction in jejunal VH due to an increase in the dietary level of CM could partly have been due to the reduction in the dietary level of digestible carbohydrates and an increase in the dietary level of fat. It could also have been due to reduced nutrient digestibility and hence luminal nutrient availability due to an increase in the dietary level of CM. CM has a greater content of fiber than SBM and corn that it replaced in the control diet. Landero et al. (2011) and Zhou et al. (2016) reported reduced energy and nutrient digestibility in weaned pigs without significant effect on growth performance due to an increase in the level of canola coproducts in diets (formulated to equal net energy and digestible nutrients) from 0% to 20%. Zhou et al. (2016) attributed the reduced energy and nutrient digestibility to the high content of fiber in CM, which dilutes dietary energy and nutrients. Feeding the increasing dietary level of CM did not affect the VH of ileum. Integrity of the upper part of small intestine compared with that of the lower part of small intestine is more affected by weaning stress and luminal availability of nutrients in pigs (Wijtten et al., 2011). The jejunum is a primary site for digestion and absorption of sugars, amino acids, and fatty acids. Thus, the lack of effect of dietary CM on ileal VH could have been due to the limited effect of dietary nutrient composition and weaning stress on ileal VH in weaned pigs.

The linear increase in the CD of ileum at the end of phase 1 due to an increase in the dietary level of CM from 0% to 40% may be explained by an increase in ileal mucosal permeability as evidenced by the decrease in ileal TEER due to the dietary inclusion of CM. The TEER reflects the opening of the tight junctions between enterocytes and hence paracellular permeability of the intestinal mucosa (Hu et al., 2013). An increase in permeability of mucosal cells in the small intestine can lead to an increase in the movement of toxins or antigens from the lumen into the small intestine (Wijtten et al., 2011). An increase in movement of toxins or antigens from the intestinal lumen into the small intestine can lead to increased secretion of mucins by cells in the crypt in an effort to protect the mucosa from toxins or antigens, leading to increased cell proliferation in the crypt and hence increased CD (Isaacson, 1998; Dupont et al., 2014).

In the current study, in vivo gastrointestinal permeability was quantified by the ratio of lactulose:mannitol concentrations in urine. Lactulose is a disaccharide, whereas mannitol is a monosaccharide. Both lactulose and mannitol are passively absorbed in the intestine of pigs but are excreted via urine because they are not metabolized within the body (Wijtten et al., 2011). Mannitol, which has a size similar to that of glucose, can be absorbed into the small intestine through the transcellular pathway or paracellular pathway, whereas lactulose, which is larger in size than mannitol because it is a disaccharide that can only be absorbed into the small intestine through the paracellular pathway (Bjarnason et al., 1995; Uil et al., 1997). Thus, it is apparent that: 1) mannitol can be absorbed into both healthy intestinal mucosa and unhealthy intestinal mucosa in which tight junctions have been damaged, whereas lactulose can only be absorbed in the unhealthy intestinal mucosa (Wijtten et al., 2011) and 2) an increase in urinary concentration of lactulose relative to that of mannitol indicates increased gastrointestinal tract permeability to toxins and pathogenic microorganisms (Bjarnason et al., 1995; Wijtten et al., 2011). In the current study, dietary CM increased the lactulose to mannitol ratio in urine, implying it increased gastrointestinal tract permeability due to the dietary CM. The increase in ratio of lactulose and mannitol in urine could partly have been due to the decreased TEER in the ileum by the increase in the dietary level of CM from 0% to 40%. The ratio of lactulose to mannitol in urine reflects the permeability of the whole gastrointestinal tract. Thus, the increase in lactulose to mannitol ratio in urine due to dietary CM could also have been due to an increase in permeability of other sections of the gastrointestinal tract such as the large intestine in which permeability was not measured in the current study.

The increase in the ratio of lactulose and mannitol in urine and a decrease in TEER in the ileum of pigs due to dietary CM are contrary to what was expected. It was assumed that dietary CM would reduce the ratio of lactulose and mannitol in urine and increase in TEER in the small intestine because of the presence of: 1) insoluble fiber in CM, which can reduce the proliferation and attachment of pathogenic microorganisms on intestinal mucosa by increasing digesta passage rate and 2) glucosinolates in CM that have antioxidant activity, implying that they can reduce oxidative stress and hence injury of intestinal mucosa. However, glucosinolates in canola coproducts can be degraded in the gastrointestinal tract into toxic products by canola seed intrinsic myrosinase or by myrosinase-like enzymes that are produced by microorganisms that reside in the gastrointestinal tract (Bell, 1993). Canola intrinsic myrosinase is heat-labile; it is inactivated at a temperature above 60 °C (McCurdy, 1992; Ludikhuyze et al., 1999; Van Eylen et al., 2007). The CM fed in the current study was produced by a prepress solvent extraction method, which involves cooking seeds before oil extraction and toasting meal after oil extraction at ≥90 °C (Newkirk and Classen, 2002), implying that the CM does not contain any active myrosinase and that glucosinolates in the CM are degraded by myrosinase-like enzymes that are produced by microorganisms that reside in the gastrointestinal tract. The toxic glucosinolate degradation products are absorbed and detoxified within the body in various organs, including the gastrointestinal tract, liver, and kidneys (Nyachoti et al., 2000; Matusheski and Jeffery, 2001; Tripathi and Mishra, 2007; Felker et al., 2016); the detoxification is initiated in gastrointestinal tract epithelial cells (Nijhoff et al., 1995; Angelino and Jeffery, 2014). Metabolism of toxic products within intestinal mucosa can lead to oxidative stress (Fishman et al., 2013; Tian et al., 2013), and oxidative stress can lead to increased permeability within the intestinal mucosa (Moser et al., 2007; Lambert, 2009). In the small intestine, microorganisms are concentrated mainly in the ileum (Gresse et al., 2019). Thus, the decrease in ileal TEER (but not jejunal TEER) due to an increase in the dietary level of CM could have been due to increased production of toxic products from glucosinolates, leading to increased oxidative stress within ileal epithelial cells and hence increased ileal permeability.

The Isc reflects net active electrogenic ion transport across the epithelium, and an increase in Isc in the small intestine mucosa can reflect either increased electrogenic cation (e.g., Na^+^) absorption or increased electrogenic anion (e.g. Cl^−^, or HCO3^−^) secretion (Wijtten et al., 2011). Dietary CM did not affect glucose-induced change in Isc, implying that dietary CM did not affect the absorptive capacity of glucose in the small intestine. Dietary CM at 20% increased growth performance and the relative abundance of Firmicutes in the feces. Lee (2019) also reported that an increase in the level of cold-pressed canola expellers in diets for weaned pigs resulted in an increase in the relative abundance of Firmicutes in the gastrointestinal tract. The relative abundance of Firmicutes in the gastrointestinal tract of humans is positively related with body weight or obesity (Guo et al., 2008; Pedersen et al., 2013), and this association has been attributed to the fact that Firmicutes increases the efficiency of digestion and absorption of energy in the gastrointestinal tract (Hildebrandt et al., 2009; Wang et al., 2019). Obesity or overweight is also positively correlated with leaky gut (Teixeira et al., 2012). In broiler chickens, the reduction in dietary level of CP resulted in increased fat content in the body (Jlali et al., 2012) and increased ileal permeability to toxins and increased expression of genes for ileal sodium-dependent glucose transport proteins (Barekatain et al., 2019). Thus, the decrease in ileal TEER due to dietary CM could also partly be associated with an increase in the relative abundance of Firmicutes in the gastrointestinal tract. However, so far, it has not been clearly established whether it is the change in the relative abundance of Firmicutes in the gastrointestinal tract that results in a change in body weight gain of animals and humans or it is a change in body weight gain of animals and humans that results in the change in the relative abundance of Firmicutes in the gastrointestinal tract.

In conclusion, the growth performance of weaned pigs was increased by an increase in the dietary level of CM from 0% to 20% and then decreased to a level similar to that for the diet with 0% CM by an increase in the dietary level of CM from 20% to 40%. However, the weights of liver and thyroid gland relative to live BW were linearly increased, whereas serum T4 level was linearly reduced by an increase in dietary level of CM from 0% to 40%. The serum IgA concentration and relative abundance of Firmicutes in feces were improved by dietary inclusion of CM at 20%. Therefore, dietary CM could be included in corn–SBM diets for nursery pigs at 20% to maximize growth performance and improve gut microbial composition and reduce immune response. Also, the CM used in the current study could be included in corn–SBM-based diets for nursery pigs at 30% or 40% without compromising growth performance. With regard to gut integrity, an increase in the dietary level of CM from 0% to 40% resulted in an increased ratio of lactulose to mannitol in the urine and a decreased TEER in the ileum, implying that the dietary CM increased gastrointestinal tract permeability partly through an increase in permeability in the ileum of weaned pigs. Thus, it appears that dietary CM at 20% improves the growth performance of weaned pigs through mechanisms other than reducing gastrointestinal tract permeability.

## Abbreviations

ADF: acid detergent fiber
ADFI: average daily feed intake
ADG: average daily gain
BW: body weight
CD: crypt depth
CM: canola meal
CP: crude protein
DM: dry matter
EE: ether extract
G:F: gain to feed ratio
IgG: immunoglobulin G
IgA: immunoglobulin A
IgM: immunoglobulin M
IL: interleukin
Isc: short-circuit current
NDF: neutral detergent fiber
PD: potential difference
SBM: soybean meal
SID: standardized ileal digestible
STTD: standardized total tract digestible
T3: triiodothyronine
T4: tetraiodothyronine
TEER: transepithelial electrical resistance
TNF-α: tumor necrosis factor-α
VH: villous height
VH:CD: villous height to crypt depth ratio
